# A Micro-Preconcentrator Combined Olfactory Sensing System with a Micromechanical Cantilever Sensor for Detecting 2,4-Dinitrotoluene Gas Vapor

**DOI:** 10.3390/s150818167

**Published:** 2015-07-24

**Authors:** Myung-Sic Chae, Jinsik Kim, Yong Kyoung Yoo, Ji Yoon Kang, Jeong Hoon Lee, Kyo Seon Hwang

**Affiliations:** 1 Center for BioMicrosystems, Korea Institute of Science and Technology, Seoul 136-791, Korea; E-Mails: bechu88@gmail.com (M.-S.C.); lookup2@hanmail.net (J.K.); yongkyoung0108@gmail.com (Y.K.Y.); jykang@kist.re.kr (J.Y.K.); 2 School of Electrical Engineering, Korea University, Seoul 136-701, Korea; 3 Department of Electrical Engineering, Kwangwoon University, Seoul 139-701, Korea

**Keywords:** DNT, gas sensor, cantilever, micro-preconcentrator

## Abstract

Preventing unexpected explosive attacks and tracing explosion-related molecules require the development of highly sensitive gas-vapor detection systems. For that purpose, a micromechanical cantilever-based olfactory sensing system including a sample preconcentrator was developed to detect 2,4-dinitrotoluene (2,4-DNT), which is a well-known by-product of the explosive molecule trinitrotoluene (TNT) and exists in concentrations on the order of parts per billion in the atmosphere at room temperature. A peptide receptor (His-Pro-Asn-Phe-Ser-Lys-Tyr-Ile-Leu-His-Gln-Arg) that has high binding affinity for 2,4-DNT was immobilized on the surface of the cantilever sensors to detect 2,4-DNT vapor for highly selective detection. A micro-preconcentrator (µPC) was developed using Tenax-TA adsorbent to produce higher concentrations of 2,4-DNT molecules. The preconcentration was achieved via adsorption and thermal desorption phenomena occurring between target molecules and the adsorbent. The µPC directly integrated with a cantilever sensor and enhanced the sensitivity of the cantilever sensor as a pretreatment tool for the target vapor. The response was rapidly saturated within 5 min and sustained for more than 10 min when the concentrated vapor was introduced. By calculating preconcentration factor values, we verified that the cantilever sensor provides up to an eightfold improvement in sensing performance.

## 1. Introduction

Many research groups have developed highly sensitive and selective sensing or detecting platforms for the explosive materials used to make immediate explosive devices (IEDs), such as trinitrotoluene (TNT), cyclotrimethylenetrinitramine (RDX), and pentaerythritol tetranitrate (PETN). These chemicals have extremely low vapor pressures at concentrations on the order of parts per million (ppm) to parts per trillion (ppt) in the atmosphere at room temperature. Detection of an infinitesimal quantity of these dangerous vapor molecules requires sensors with high sensitivity, high accuracy, and rapid response. Methods for improving the sensitivity and response time, such as the use of a high affinity target molecule receptor and additional pretreatment techniques, have been employed in previous sensor systems.

Improvements in micro/nano electromechanical systems (MEMS/NEMS) technology have led to the development of various miniaturized mechanical sensors for chemical and biological applications requiring high sensitivity. The cantilever sensor is the most widely used promising micromechanical sensor owing to its excellent sensing capabilities [1,2]. The detection limit of the cantilever sensor is approximately 500 ppt, which is sufficient for detecting gas vapors with very low concentration [3]. Cantilevers that are capable of real-time measurement also have short response time, though the response time depends on the reaction between target molecules and the receptors.

Receptors such as antibodies, deoxyribonucleic acid/ribonucleic acid (DNA/RNA) aptamers, or peptides that have a binding specificity make it possible to selectively immobilize the target molecules on the surface [4,5]. These specific binding interactions between receptors and target molecules on the sensor surface change the mechanical properties of the cantilevers, and these changes can be observed by analyzing the resonant frequency response in the dynamic mode [6]. Resonant frequency shifts verify the mass loading of specific interactions on the sensor surface. Generally, the mass loading of target molecules is the dominant variable in relation to changing the resonant frequency. A dynamic cantilever is preferable in terms of quantitative analysis for small molecules owing to its robustness against external disturbance and noise [7,8]. Measuring the resonant frequency change in the dynamic mode of a cantilever sensor is effective also for chemical-vapor monitoring [3,9].

Although cantilever sensors provide sufficiently high sensitivity for detection of extremely low vapor pressures in chemical gas-mixture sensing applications, such as when explosive or volatile organic compounds (VOCs) in the atmosphere must be detected, it is possible to further enhance the sensing performance. This enhancement can be achieved using a preprocessing procedure for the sample, such as a preconcentration step, in order to improve sensitivity and the detection limit. Preconcentration is commonly used as a preprocessing tool for gas-mixture analysis; it improves quantitative analytic performance by trapping analytes and releasing them into a detector as an enriched plug. Using MEMS fabrication technology, it is possible to develop a compact micro-preconcentrator (µPC) that achieves high concentration efficiency [10,11].

Here, we introduce a cantilever-based chemical-vapor detection system combined with a µPC for detecting ultra-low concentrations of 2,4-dinitrotoluene (2,4-DNT) vapor, which is a by-product and trace marker of the explosive molecule TNT. The 2,4-DNT molecule exists in concentrations of 160 parts per billion (ppb) at room temperature at atmospheric pressure. The fabricated microcantilever sensor is operated by an embedded piezoelectric thin film. The specific sequenced 12-mer peptides were immobilized as receptors for 2,4-DNT vapor on the cantilever surfaces to provide the selectivity of the cantilever sensor. The µPC was also fabricated using the MEMS techniques, and it was coated with an adsorbent polymer to provide structures for trapping volatile explosive-related compounds. The µPC was incorporated ahead of the sensing reaction chamber, where it can accumulate and release target vapor. The volume of the sample matrix and environmental conditions for preconcentration were established by considering compatibility with our cantilever sensor. Comparing the resonant frequency change of cantilever sensors with and without a sample preconcentrator confirmed the expected performance enhancement.

## 2. Experimental Details

**Figure 1:**
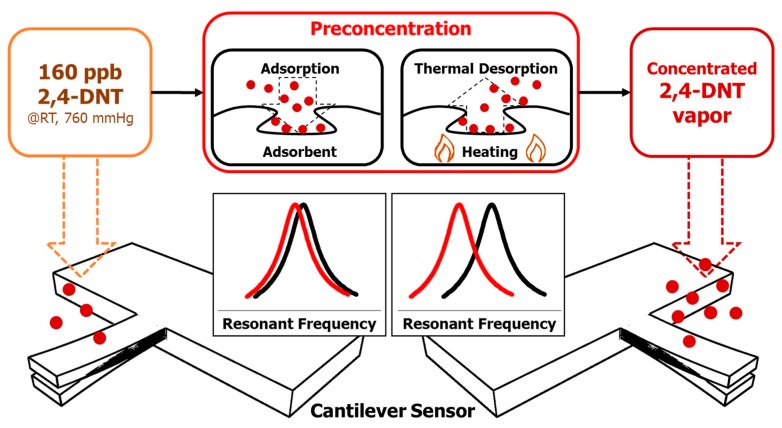
Functional model of the cantilever-based artificial olfactory system with sample preconcentration for 2,4-DNT vapor detection.

A diagram representing the 2,4-DNT gas-vapor detection protocol with the proposed µPC-integrated cantilever sensor system is provided in Figure 1. The surface of the cantilever was functionalized by 12-mer peptide receptors for high binding specificity resulting in high selectivity. The binding interaction of 2,4-DNT vapor leads to changes in the resonant frequency of the dynamic actuated cantilever sensor, and the change in resonant frequency is measured. The integrated µPC concentrated the target gas vapor, 2,4-DNT, using Tenax-TA. The concentrated 2,4-DNT gas was desorbed with 270 °C thermal heating and flowed into the reaction chamber containing the cantilever sensor. The changes in resonant frequency were measured using a real-time electrical detection system.

**Figure 2:**
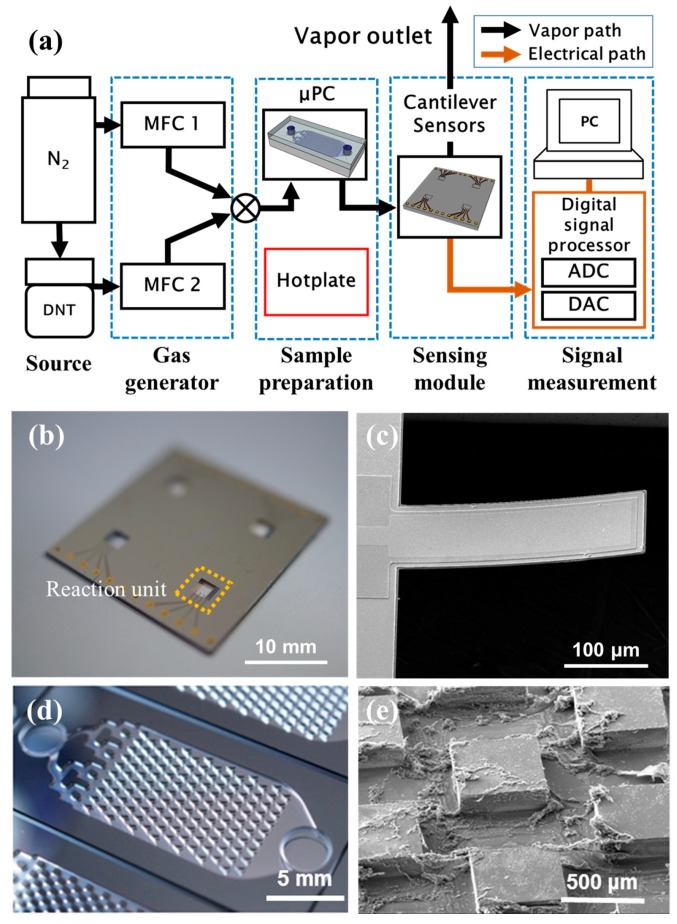
Olfactory sensing system integration with micromechanical cantilever sensors and μPC for detection of 2,4-DNT vapor (**a**) Schematic of the experimental setup for gas vapor detection with sample preconcentration. (**b**) Fabricated multiarrayed cantilever sensor with four reaction units. Each unit includes three operable cantilevers. (**c**) Multilayered single cantilever with dimensions of 100 × 300 μm. (**d**) Fabricated Si-based μPCs with microstructures. (**e**) SEM image of coated adsorbent (Tenax-TA) on the microstructure of μPC.

The detection system includes a multiarrayed cantilever sensor, a gas-generating system with a µPC, and a signal-processing system, as shown in Figure 2a. The gas-generating system consists primarily of a pair of mass-flow controllers (MFCs), solenoid valves, and an on/off toggle switch. Continuously injecting nitrogen gas (N_2_) at a 50 standard cubic centimeter per minute (sccm) flow rate provided stabilization of the resonant frequency and 2,4-DNT molecule delivery. The cantilever sensor was located in a reaction chamber connected between the gas-generating and signal-processing systems. The reaction chamber has an inlet and outlet through a sensor chip loading for a gas sample stream that was prepared from polyetheretherketone (PEEK) material [9]. The µPC was located in front of the reaction chamber containing the cantilever sensors, and it was placed on a heater to maintain the thermal desorption temperature. Then, we connected a signal-processing system for measuring the sensor’s resonant frequency. The signal-processing system enabled real-time monitoring of changes in the resonant frequency of the sensor due to interactions between the target vapor and the peptide receptors. For measuring electrical signals from the cantilever array, we employed a PC-controlled electrical measurement system (MiCan 2.0, Cantis Corporation, Ansan, Korea) equipped with an analog-to-digital converter (ADC), a charge amplifier, a digital signal processor (DSP), and a digital-to-analog converter (DAC).

We fabricated a multiarrayed micromechanical cantilever sensor with 2.18-µm-thick SiN_x_/Ta/Pt/PZT/Pt/SiO_2_ multilayers (see Figure 2b). In order to actuate the cantilever, 1.5-µm-thick piezoelectric layer (PZT) was deposited via the sol-gel method; it was intended to function as a self-actuating layer without need for external oscillating equipment. We thus fabricated a dynamic mode cantilever that offers higher Q-factor and sensitivity than a static mode cantilever. According to Equation (1), the resonant frequency (*F_r_*) is determined not only by the inducing mass and surface stress, caused by molecular interaction on the cantilever surface, but also by the thermal events, which can affect the mechanical characteristics owing to the different thermal expansion coefficients of each layer [12,13,14]:

(1) $$Fr=\frac{1}{2\pi }\sqrt{\frac{k}{m^{*}}}$$

where *k* is the spring constant and m^*^ is the effective mass of the cantilever. In our experimental methods, thermal issues could occur in the 2,4-DNT preconcentration process. A dynamic mode cantilever sensor with embedded piezoelectric actuating layer was designed to avoid this subsidiary effect in order to achieve accurate sensing of low-concentration gas vapors.

The fabrication process for the multi-arrayed cantilever sensors followed that used in previous studies [9,15]. The fabricated sensors have four reaction units (indicated by the yellow-dashed-line rectangular area in Figure 2b), and each unit contains three individual cantilevers that operate as an array and a reference cantilever that ensures accurate resonant frequency measurement by reducing parasitic capacitance. An example of the three operable cantilevers in the reaction unit is shown in Figure 2c.

The fabricated piezoelectric cantilever sensors have resonant frequencies of 24.5 ± 3 kHz and dimensions of 100 × 300 µm, and these values are uniform for all the cantilevers in a reaction unit array. To avoid external disturbance during interactions between the target vapor and the peptide receptor on the cantilever sensor surface, the four reaction units can be functionalized for different surface conditions (2,4-DNT specific peptide, 2,4-DNT nonspecific peptide immobilized and peptide-receptor-free surface).

A Si-based µPC was fabricated and sealed with a Pyrex glass cover consisting of a gas inlet and outlet and a concentration chamber with dimensions of 10 × 20 × 0.3 mm (width × length × thickness) (see Figure 2d). The concentration chamber has a micropattern that increases the surface area for gas-vapor adsorption. Sandblasting was used to form inlet and outlet holes for gas collection on the Pyrex glass wafer, and the microstructural Si wafer was bonded with the Pyrex glass wafer via anodic bonding. For the 2,4-DNT concentration used as a sample pretreatment, polymer adsorption and thermal desorption phenomena occurring between the adsorbent and specific molecules were utilized according to a gas-collection method [16]. Tenax-TA is a 2,6-diphenylene oxide-based porous polymer resin. Its capabilities have been validated for sample treatment in gas analysis instruments, such as gas chromatograph-mass spectrometers (GC-MS). Coating the concentration chamber with Tenax-TA (80–100 mesh, Sigma-Aldrich, St. Louis, MO, USA,) makes it possible to selectively preconcentrate the volatile organic analytes of interest [17,18]. Tenax-TA adsorbent was dissolved in dichloromethane with a concentration of 10 mg/mL. The microstructures were immersed in an adsorbent solution and washed with methyl alcohol. After evaporation of the solvent, Tenax-TA was deposited as shown in Figure 2e.

The peptide receptors were immobilized on the bottom surface of the cantilevers. The specific binding peptide (SBP) for the target vapor has a sequence (12-mer, His-Pro-Asn-Phe-Ser-Lys-Tyr-Ile-Leu-His-Gln-Arg) determined by phage-display screening to bind 2,4-DNT with high affinity [3]. The non-specific binding peptide (NSP) has randomly allocated sequences (12-mer, Thr-Ser-Met-Leu-Leu-Met-Ser-Pro-Lys-His-Gln-Ala) and was immobilized as a control on another cantilever array. At first, the Au layer (50 nm) was deposited with a Cr adhesive layer (10 nm) on the rear surface of the cantilever using an e-beam evaporator. The Au surface was cleaned with piranha solution (a 4:1 ratio of H_2_SO_4_ to H_2_O_2_) and rinsed with deionized (DI) water to remove potential contaminants. Self-assembled monolayers (SAMs) were formed on the Au surface by immersing a mixed solution with a 9:1 ratio of tri-(ethylene glycol)-alkanethiol (HS-EG_3_-OH) to carboxyl-terminated penta-(ethylene glycol)-alkanethiol (HS-EG_6_-COOH) in ethanol for 12 h. The modified Au surface was treated with a mixture of 1-ethyl-3-(3-dimethylaminopropyl) carbodiimide (EDC) and 2,5-pyrroledione (maleimide) dissolved in a phosphate buffered solution (PBS, pH = 7.4) with 100 mmol/L and 50 mmol/L, respectively, for 2 h. A peptide-receptor solution with a concentration of 10 µg/L in a PBS was incubated on the surface of the maleimide-functionalized SAMs for 2 h. The cysteine terminal from the thiol group of peptides was conjugated with maleimide to form a thioether linkage. The surface was cleaned with PBS, DI water, and ethyl alcohol for each process.

## 3. Results and Discussion

**Figure 3:**
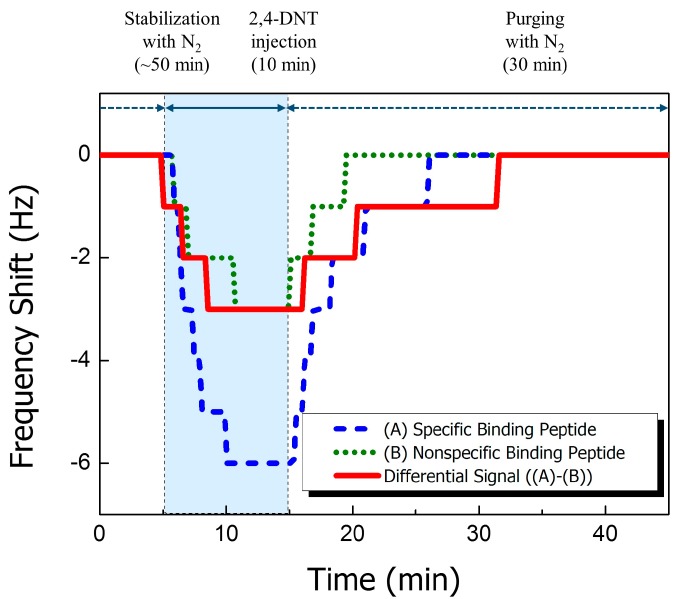
2,4-DNT differential frequency responses (red solid line) between cantilevers functionalized with specific binding peptide receptors (blue dashed line) and nonspecific binding peptide receptors (green dotted line) in an environment containing 160 ppb 2,4-DNT.

The sensitivity and selectivity of the cantilever sensors were verified without preconcentration by injection of 160 ppb of 2,4-DNT vapor mixed with N_2_ gas for 10 min after stabilization with 50 sccm of N_2_ gas for 50 min. After a reaction time of 10 min, the reaction chamber was purged with pure N_2_ at 50 sccm. The total volumetric stream of 2,4-DNT vapor was 500 mL. The resonant frequency was immediately changed after target-vapor injection, as shown in Figure 3. The response of the SBP (blue dashed line) and NSP (green dotted line) immobilized cantilever sensors was −6 Hz and −3 Hz, respectively. This 100% increase in the resonant frequency shift shows that SBP has a higher binding affinity than NSP. The change from net binding reactions (red solid line) was only −3 Hz. The values were measured via the differential frequency shift of SBP and NSP, and the difference in resonant frequency was saturated and stabilized within 5 min. After purging with N_2_ gas, the resonant signal recovered to its normal state. The changes demonstrate that the binding affinity of the peptide receptor leads to resonant frequency changes that are caused by 2,4-DNT gas vapor and that the resonant frequency returns to its normal state when the target gas is removed. Our previous study demonstrated similar frequency shift trends for this differential signal [9].

Prior to evaluating sample preconcentration with a cantilever sensor, it is necessary to define the thermal effects on a multilayered cantilever sensor. Basically, the different volumes of 2,4-DNT vapor were collected via direct injection into the µPC with constant 50 sccm volumetric flow and released via thermal desorption to form a plug stream. The adsorption of the 2,4-DNT is an exothermic chemical reaction that occurs at room temperature with adsorbent. In order to form a highly enriched 2,4-DNT plug, the µPC with adsorbent was rapidly heated by a heater set to 270 °C to achieve an endothermic desorption process. When the heated 2,4-DNT plug is introduced into the sensor with 50 sccm N_2_ gas, thermal issues can cause frequency fluctuations in the multilayered cantilever sensors by affecting the difference in the thermal expansion coefficients of each layer. Thus, the heating temperature and time for thermal desorption were optimized to avoid this signal interference. To investigate responses due to thermal effects, the cantilever sensor was heated directly from room temperature to 50 °C. The resonant response indicated that our multilayered cantilever has a temperature dependence of 4.6 Hz/°C, as shown in Figure S1 (see Supplementary Information). In regard to eliminating the thermal effects of the endothermic process in preconcentration, we determined that maintaining the sensor at 270 °C for a minimum of 2.5 min with a 50 sccm injection of pure N_2_ gas would sustain stable operation and satisfy the thermal desorption conditions (see Figure S2).

**Figure 4:**
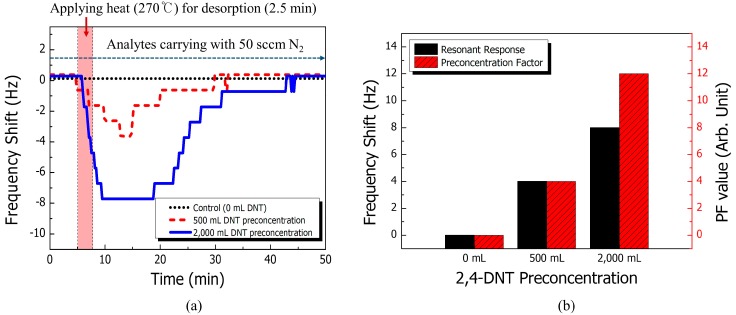
Performance evaluation for sample preconcentration. (**a**) Resonant response with 0, 500, and 2000 mL 2,4-DNT vapor preconcentration for the cantilever sensor with negative control. (**b**) Frequency shift and preconcentration factor for each preconcentration condition.

Next, resonant frequency shifts according to the volumes of 160 ppb 2,4-DNT vapor collection (500 mL and 2000 mL) were measured to evaluate the performance enhancement of the cantilever sensor with sample preconcentration. As shown in Figure 4a, differential signals between SBP and NSP arrays of 500 mL and 2000 mL were obtained as −4 Hz and −8 Hz, respectively. With a preconcentration of 500 mL 2,4-DNT, the resonant frequency decreased for 10 min after sample desorption and recovered under continuous N_2_ flow. A 2,4-DNT volume of 2000 mL is sufficient to compensate for potential physisorption and allows for fully saturated trapping in a µPC in the limited space of the reaction chamber. The signal for preconcentrated 2,4-DNT vapor with a volume of 2000 mL decreased more dramatically than in the environment with lower preconcentration. Whereas the volume of 2,4-DNT vapor collected was four times larger, the signal response doubled compared to previous experiments. The signal was rapidly saturated within 3.5 min via thermal desorption of the concentrated sample, and the saturated condition was sustained for 10 min. Then, the area under the differential signal was four times larger than with the 500 mL preconcentration condition. This provided more time for recovering the resonant responses. These resonant responses indicate that a higher concentration of 2,4-DNT vapor than is found in the atmosphere was successfully formed and carried into the functionalized cantilever arrays. Based on the response in an environment containing 160 ppb of 2,4-DNT without the µPC, the estimated concentration of preconcentrated 2,4-DNT vapor was 213 ppb and 426 ppb for sampling volumes of 500 mL and 2000 mL, respectively. This shows that more binding interaction occurred in the larger quantitative molecules in the preconcentration environment. These concentration values were used to determine the preconcentration factor (PF). The preconcentration performance is generally defined as:

(2) $$PF=\frac{C_{f}}{C_{0}}$$

where *C*_0_ is the concentration of analytes before sample pretreatment and *C_f_* is the concentration of preconcentrated analytes. The PF is commonly used as a parameter for evaluating µPC performance as determined by experimental conditions and detectors. The PF value indicates the signal amplification in the detector based on preconcentration with conventional gas chromatography techniques [19,20]. Therefore, we used the ratio of the maximum concentration of trapped 2,4-DNT vapor and the concentration of normally injected target vapor without preconcentration. In a previous study, the concentration was estimated based on the linear relationship between 2,4-DNT concentration and frequency shift [3]. The sample volume for calculating the concentration was determined by the thermally desorbed 2,4-DNT vapor. The PF values for the 500 mL and 2000 mL sample volume were calculated as 4 and 12, respectively (see Figure 4b). The trend of the resonant frequency response follows that of the PF values according to volume, as shown in Figure 4b.

**Figure 5:**
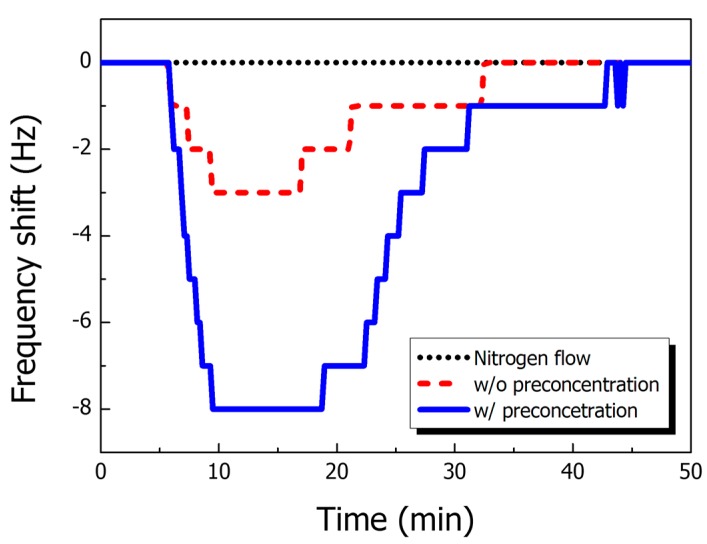
Relative signal response with different sample preprocessing environments: pure N_2_ flow (black dotted line), direct injection of 160 ppb 2,4-DNT (red dashed line), and 2,4-DNT sample preprocessing with µPC (blue solid line).

Figure 5 shows the effect of the preconcentrator in DNT detection with real-time monitoring. The resonant frequency shift occurred with 160 ppb 2,4-DNT gas for both cases (with and without preconcentration). However, the resonant frequency change with preconcentration is clearly improved relative to that without preconcentration under the same real-time monitoring conditions; the frequency change was approximately three times larger. The calculated PF values suggest that the sensing performance of a cantilever sensor can be enhanced eightfold relative to what was achieved in previous research. Although there are some differences between our theoretical approaches and experimental results, the enhancements are clearly demonstrated, and the system can achieve a sub-ppt detection limit.

## 4. Conclusions

In this paper, we confirmed the enhanced gas-molecule detection sensing performance with 2,4-DNT vapor molecules can be achieved by equipping cantilever sensors with a µPC. Combining these two microfabricated devices makes it possible to develop highly sensitive and selective miniaturized gas-analysis systems. Prior to the sample pretreatment evaluations, real-time monitoring of the resonant frequency verified that immobilizing the specific binding peptide on the surface of a micromechanical cantilever sensor provides accurate discrimination between the target molecule and a nonspecific binding peptide. We demonstrated that our µPC is effective, and our cantilever detected 2,4-DNT vapor and exhibited enhanced sensing performance. We used a µPC with an adsorbent coating for sample pretreatment and located it in front of the cantilever sensor to collect and release 2,4-DNT vapor, and this approach tripled the amplitude of the differential signal. The system was able to detect concentrations as low as several hundreds of ppb. This sensitivity is similar to or lower than those reported by other researchers [21,22]. However, we can achieve higher sensitivity by more effectively optimizing the gas flow systems and the condition for releasing 2,4-DNT after preconcentration. For practical applications, the size of the system and detection performance in realistic atmospheric conditions must be considered [21,23]. We anticipate that the techniques described in this paper will make it possible to improve the conventional analytic instruments used in laboratories and will be employed in the field in the near future.

## Supplementary Files

Supplementary File 1
