# Supplementary material for: A Micro-Preconcentrator Combined Olfactory Sensing System with a Micromechanical Cantilever Sensor for Detecting 2,4-Dinitrotoluene Gas Vapor
Source: Sensors (Basel). 2015 Jul 24;15(8):18167–77. doi: 10.3390/s150818167 (PMC4570313; doi:10.3390/s150818167)
Supplement: Supplementary File 1 [file sensors-15-18167-s001.pdf]

OPEN ACCESS
sensors
ISSN 1424-8220
www.mdpi.com/journal/sensors

Supplementary Information

A Micro-Preconcentrator Combined Olfactory Sensing System with a Micromechanical Cantilever Sensor for Detecting 2,4-Dinitrotoluene Gas Vapor. *Sensors* 2015, *15*, 18167-18177

Myung-Sic Chae [1,2,†], Jinsik Kim [1,†], Yong Kyoung Yoo [1,3], Ji Yoon Kang [1], Jeong Hoon Lee [3,*] and Kyo Seon Hwang [1,*]

[1] Center for BioMicrosystems, Korea Institute of Science and Technology, Seoul 136-791, Korea; E-Mails: bechu88@gmail.com (M.-S.C.); lookup2@hanmail.net (J.K.); yongkyoung0108@gmail.com (Y.K.Y.); jykang@kist.re.kr (J.Y.K.)
[2] School of Electrical Engineering, Korea University, Seoul 136-701, Korea
[3] Department of Electrical Engineering, Kwangwoon University, Seoul 139-701, Korea

† These authors contributed equally to this work.

* Authors to whom correspondence should be addressed; E-Mails: jhlee@kw.ac.kr (J.H.L.); kshwang@kist.re.kr (K.S.H.); Tel.: +82-2940-8372 (J.H.L); +82-2958-6727 (K.S.H.).

S1. Frequency Shift Depends on Temperature

In order to evaluate the effect of a thermally induced environment, a multilayered cantilever sensor was heated with a hotplate from room temperature (25 °C) to 50 °C. The relationship between resonant frequency and temperature was calculated as 4.6 Hz/°C.

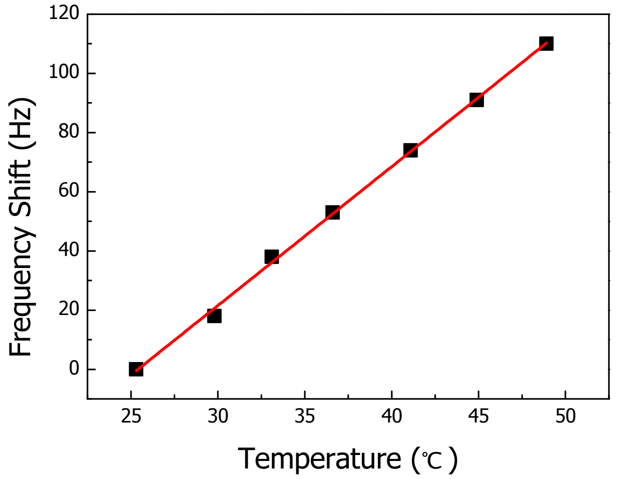

Figure S1. Linear relationship between temperature and resonant frequency (4.6 Hz/°C).

S2. Optimization for Thermal Desorption with μPC

If Tenax-TA resin is used as the adsorbent for the preprocessing of various aromatic nitro compounds or volatile organic compounds, it is known that the desorption temperature is required to be greater than 200 °C with rapid heating when using conventional gas-analysis equipment (e.g., Gas chromatography-flame ionization detector (GC-FID)). In order to eliminate unexpected frequency changes caused by thermal desorption in the environment that precedes the cantilever sensor, it is necessary to determine the temperature and the heating time. When the cantilever is exposed to sufficient thermal desorption conditions (270 °C for 2.5 min) with pure 50 sccm N_2, the resonant frequency of all arrays with differently functionalized surfaces stabilizes within 40 min after the heating process. Figure S2b and S2c present the resulting fluctuations with heating times of 5 min and 10 min, respectively.

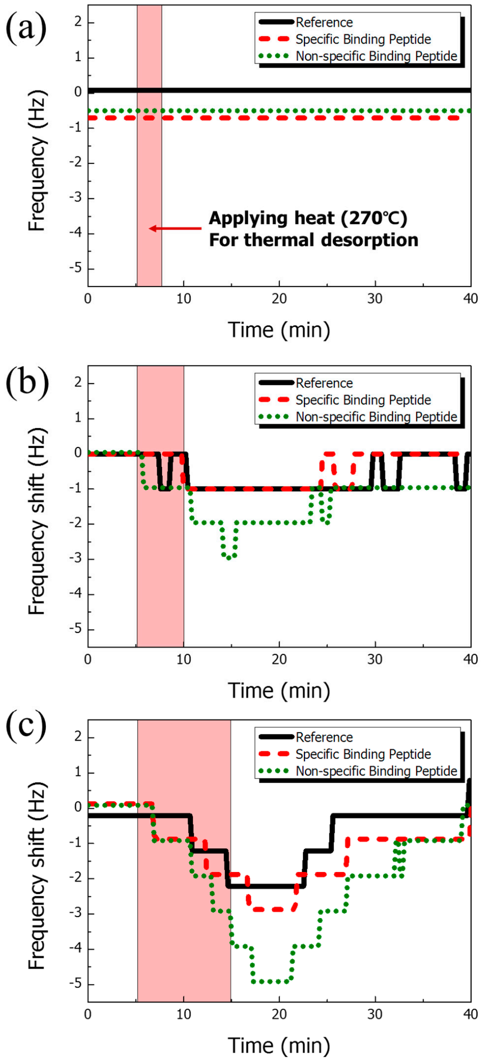

Figure S2. Stability of resonant signals in an optimized thermal desorption environment. A temperature of 270 °C was rapidly applied for (**a**) 2.5 min. (**b**) 5 min. (**c**) 10 min with injection of 50 sccm of pure N_2 gas to each cantilever array.

© 2015 by the authors; licensee MDPI, Basel, Switzerland. This article is an open access article distributed under the terms and conditions of the Creative Commons Attribution license (http://creativecommons.org/licenses/by/4.0/).
